# Delirium—biomarkers and genetic variance

**DOI:** 10.3389/fphar.2014.00075

**Published:** 2014-04-16

**Authors:** Nicoleta Stoicea, Sean McVicker, Alexander Quinones, Priscilla Agbenyefia, Sergio D. Bergese

**Affiliations:** ^1^Department of Anesthesiology, Wexner Medical Center, Ohio State UniversityColumbus, OH, USA; ^2^College of Arts and Sciences, The Ohio State UniversityColumbus, OH, USA; ^3^College of Medicine, The Ohio State UniversityColumbus, OH, USA; ^4^Department of Neurological Surgery, Wexner Medical Center, Ohio State UniversityColumbus, OH, USA

**Keywords:** delirium, miRNA, APOE ε4, neuroinflammation, cholinesterase activity dopamine receptors, S-100B

## Introduction

Advancement in quality of health care has resulted in improved patient outcomes; however, knowledge of delirium as well as methods of treatment and its prevention are not well known. The prevalence of delirium in postoperative patients is associated with an increase in mortality, impaired recovery, and increased hospital costs (Chu et al., 2011). Complex syndromes, like delirium, are usually not associated with a single independent cause but instead with a number of different sources (van Munster et al., 2011). Delirium is defined by the *Diagnostic and Statistical Manual of Mental Disorders*, fifth *edition* (DSM-V) as a disturbance in attention and awareness according to several criteria (American Psychiatric Association, 2013). Biomarkers are biochemical or molecular traces related to the presence or severity of a disease (Chu et al., 2011). Biomarkers present before the onset of the disease can be used as a risk marker, the rise and fall of biomarkers during illness can be tracked as a disease marker, and remaining biomarkers can be seen as an end product of a disease (Chu et al., 2011). The aim of this paper is to identify biomarkers that may have a diagnostic and prognostic role in delirium.

## Neuroinflammation

There are many potential inflammatory serum and genetic biomarkers to investigate along with delirium and PD. However, it may not be sufficient to simply analyze serum inflammatory proteins; a 2006 review noted multiple problems with using inflammatory serum biomarkers as a primary measure of inflammatory reaction and delirium association, including the large number of inflammatory cascade proteins involved (levels of each can vary on an individual basis) and the fact that the cytokine response can be confounded by many factors (age, type of insult, comorbid conditions, etc.) (Munster et al., 2011). A more promising strategy may investigating delirium in conjunction with the genetics underlying an individual's inflammatory response. A study investigating Interleukin-6 (IL-6), Interleukin-8 (IL-8), and Interleukin-6 Receptor (IL-6R) genetic variations and delirium, found no polymorphisms associated with delirium (Marcantonio et al., 2006; van Munster et al., 2011). However, a preliminary case-control investigation by the same research group found a link between human brain activity of microglia, astrocytes and IL-6 and delirium, with elevated HLA-DR, CD68, IL-6 being associated with an increased prevalence of delirium, and the biomarkers GFAP and IL-1β showing no association (Munster et al., 2011). These results suggest the possibility that there could be an underlying mechanism associated with brain-localized inflammation precipitating delirium, a conclusion warranting further investigation.

## Dopamine receptors

Genetic variance in genes encoding dopamine receptors has been studied in possible relation to delirium. Genotyping performed on DNA isolated from 720 hip fracture elderly patients concluded that AA genotype of the SLC6A3 gene (rs393795) is protective for the development of delirium while genetic polymorphisms in the receptor DRD2 and dopamine transporter genes, were associated with delirium (rs6276, rs6277, and rs2734839) (Munster et al., 2011). All seven SNPs in the SLC6A3 gene were associated with delirium; the gene affects the function of dopamine transporters, effectively reducing the concentration of cerebral basal dopamine and increasing delirium risk (van Munster et al., 2010c). The rs6276 SNP has been associated with increased alcohol use, which might be due to a lower production rate, resulting in less D2 protein (Lucht et al., 2007). The rs6277 SNP affects messenger RNA (mRNA) stability and results in altered dopamine-induced up regulation of DRD2 expression and DRD2 mRNA translation (van Munster et al., 2010c).

A further meta-analysis concluded that the homozygous AA genotype of rs393795 in the SLC6A3 gene was more frequent in patients without delirium, and the homozygous GG genotype of rs6276 in the DRD2 gene was more frequent in patients without delirium after adjustment for cognitive impairment (van Munster et al., 2010b). The SLC6A3 gene could affect availability or function of dopamine transporters; lower cerebral basal dopamine concentration could diminish risk for delirium (van Munster et al., 2010b). The rs393795 SNP is intronic, and intronic sequences have been found to encode microRNAs; future research could involve sequencing this region for functional variants (van Munster et al., 2010b).

## S-100B and neuron specific enolase

Biomarkers of cerebral damage could also potentially be used as a biomarker of PD. S100B is elevated in blood and cerebrospinal fluid (CSF) when nervous system damage occurs as a result of cerebral damage (van Munster et al., 2009b). A postoperative elevation in S100B levels in elderly hip fracture repair patients has been observed in conjunction with PD along with elevated postoperative IL-6 and IL-8 serum concentrations in the same population (van Munster et al., 2009b). However, no link between IL-6 and IL-8 genetics and delirium has yet been found (van Munster et al., 2011). Interestingly, Olivecrona and Koskinen found in 2012 that traumatic brain injury (TBI) patients with the APOE ε 4 allele exhibited significantly higher maximal levels of the S-100B protein than those without the allele suggesting that there is a genetic susceptibility to increased levels of S-100B following a neurologic insult (TBI in this case) (Olivecrona and Koskinen, 2012). No similar study has been conducted to examine the interplay of genetics and S-100B in PD, so it is a potential area for future research. In this same study, Olivecrona and Koskinen, found that neuron specific enolase (NSE) was also elevated in TBI patients with the Alzheimer's disease related APOE ε 4 allele compared to those without (Olivecrona and Koskinen, 2012). NSE has also been examined in patients with PD, but no significant correlation between elevated levels of NSE and PD has yet been found (van Munster et al., 2009b).

## APOE ε4

Genetic variations in the apolipoprotein E have been studied in relation to delirium. A study done by Ely et al. including mechanically ventilated critically ill patients, examined the APOE4 polymorphism and its relationship with delirium and discovered a strong association between the APOE4 polymorphism and a longer duration of delirium (Ely et al., 2007). APOE4 is less effective at suppressing nervous system inflammation (Ely et al., 2007). Another study, surveying various topics relating genetics and delirium noted the APOE4 genotype is associated with increased inflammation in animal models, and that cytokines can cause a reduction in the acetyl cholinergic pathways (van Munster et al., 2009a). Another article summarizing the known biomarkers for delirium notes on the inconsistencies in the outcomes of APOE E4 studies (Chu et al., 2011). The lack of notification of pre-existing cognitive impairment in most studies prevents definite association between the allele and delirium (van Munster et al., 2009a).

## Hypothalamic-pituitary-adrenal (HPA) axis

The neuroadrenergic axis has been a prominent focus of research on the etiology of PD. PD is associated with elevated cortisol and less frequent suppression of IGF-I (Cerejeira et al., 2013). Similarly, delirium is associated with higher levels of cortisol in CSF measurements (Pearson et al., 2010). Hip fracture patients who were homozygous for BclI-TthIIII haplotype (a haplotype associated with increased sensitivity to glucocorticoids and elevated diurnal cortisol levels) were at a decreased risk of developing PD (Manenschijn et al., 2011). One study found no association between plasma glucose and delirium, but did take note of the hyperactivity of the HPA axis along with high plasma and CSF cortisol levels in patients with delirium compared to those without (Bisschop et al., 2011). This same study notes that delirium has been reported in patients with Cushing's syndrome (characterized by excess cortisol), as well as in those undergoing glucocorticoid therapy (Bisschop et al., 2011). Another study focused on the association between serum composition and delirium noted that HPA dysregulation in delirium resulted in higher basal cortisol levels associated with chronic cognitive impairment and higher levels of cortisol under conditions of acute stress; they concluded that these acute elevations of cortisol could be involved in development of delirium (van Munster et al., 2010a).

## Ischemia

PD is a prominent cause of morbidity following hip arthroplasty, and cerebral fat emboli often occur during the surgical process—investigators have identified the resulting cerebral ischemia as a potential link worthy of investigation (Cox et al., 2011).

There is extensive research on the genetic biomarkers of cerebral ischemia. MicroRNA-124 (miRNA-124) has been reported to regulate differentiation of neural progenitor cells (Cheng et al., 2009). In rats, a decrease in neurogenesis following an induced ischemic stroke was associated with decreased miRNA-124 expression (Liu et al., 2011). There is evidence that a reduced expression of miRNA-124 results in decreased Ku70 expression and neuronal death following ischemia and reperfusion injury (Zhu et al., 2014). Similarly, there is also evidence that miRNA-124 plays a role in down regulating inflammatory cytokines (often indicated in the pathogenesis of PD) (Deiner and Silverstein, 2009; Sun et al., 2013). Further research could elucidate a possible link between the miR-124 molecular pathway and PD. There are other miRNA associated with ischemic stroke and subsequent neurogenesis, but they are not studied as in depth as miR-124 (Yan et al., 2013).

## Serum anticholinergic activity

Serum anticholinergic activity has also been implicated in the pathogenesis of PD: acetylcholinesterase (Ach) and butyrylcholinesterase (BuChE) are involved in the production of inflammatory cytokines, and are hypothesized to play a role in PD development through this mechanism. One study found significantly decreased activity of these enzymes both preoperatively and postoperatively in patients experiencing PD (Cerejeira et al., 2012). Another study found a significant link between lower cholinesterase activity and increased inflammatory markers (C-reactive protein, IL-6) (Cerejeira et al., 2011). The finding of decreased preoperative cholinesterase activity in PD patients suggests that cholinesterase activity could potentially be a predisposing factor for PD. Cerejeira et al. mentioned that the preexisting difference in anticholinergic activity could be due to pharmacologic therapy, as well as overall health, and genetic differences (Cerejeira et al., 2012). The cholinergic system is associated with a wide variety of physiological processes in the brain that could alter cognitive function (Cerejeira et al., 2010). There are many polymorphisms in the cholinesterase gene (Goodall, 2004). Cholinergic physiology has been implicated in the pathophysiology of multiple neurodegenerative disorders (AD, Parkinson's) and inflammation related diseases, and the complex genetics (including both genetic polymorphisms and mi-RNA regulation) of the cholinergic system have been proposed as a potential area for identifying biomarkers of these disorders (Shenhar-Tsarfaty et al., 2013). Cholinesterase activity has been associated with PD, so the investigation of biomarkers of cholinergic system dysfunction in conjunction with PD is warranted.

## Conclusions

The pathogenesis of PD is still poorly understood. Further elucidation and refinement of biomarkers to track the risk and progression of this disorder would help in the identification and treatment of patients at risk for this devastating disorder. Future research should focus on both identifying serum markers of delirium and understanding the genetic basis behind these processes. Hopefully, these efforts will make important strides in improving outcomes of patients experiencing delirium.

### Conflict of interest statement

The authors declare that the research was conducted in the absence of any commercial or financial relationships that could be construed as a potential conflict of interest.
